# A follow-up study for biomass yield QTLs in rice

**DOI:** 10.1371/journal.pone.0206054

**Published:** 2018-10-23

**Authors:** Kazuki Matsubara, Jun-ichi Yonemaru, Nobuya Kobayashi, Takuro Ishii, Eiji Yamamoto, Ritsuko Mizobuchi, Hiroshi Tsunematsu, Toshio Yamamoto, Hiroshi Kato, Masahiro Yano

**Affiliations:** 1 Institute of Crop Science, NARO (National Agriculture and Food Research Organization), Tsukuba, Ibaraki, Japan; 2 National Institute of Agrobiological Sciences, Tsukuba, Ibaraki, Japan; University of Guelph, CANADA

## Abstract

The biomass yield (plant weight) of rice fluctuates from year to year. In a previous study, we demonstrated that six quantitative trait loci (QTLs) contribute to the variation in the plant weight of recombinant inbred lines (RILs) of high-yielding Japanese rice cultivars. However, it remains unclear whether the effects of those QTLs are stable over multiple years. Therefore, we evaluated the effect of the alleles on the plant weight of RILs over multiple years, including a change of fertilization level (i.e., in different environments). Even though the biomass yields of all RILs fluctuated among environments, RILs that were selected on the basis of the genotypes of the detected QTLs had a stable rank order of plant weight that corresponded to their genotypes. This multiple-environment experiment reveals the highly significant contribution of both genotypic and environmental variances to the observed variance in plant weight. A marginally significant QTL–environment interaction was detected at only one of the six QTLs, with a subtle contribution. These results support the idea that the biomass yield of rice can be improved through QTL-based allele selection.

## Introduction

Rice (*Oryza sativa* L.) is the staple food of more than half of the world’s population [1], and in areas where populations continue to grow [2, 3], further improvement in grain yield has become a major challenge for rice breeders and geneticists. In South Korea and Japan, rice is also used for livestock feed, which is used not only as a grain but also as whole-crop silage (WCS). Therefore, biomass yield has been an important target trait in rice-breeding programs in these countries.

In general, the effect of artificial selection on specific traits depends on the genetic architecture of the target species. For example, the yields of crops, such as maize [4], rice [5, 6], and wheat [7], are complex quantitative traits that involve multiple quantitative trait loci (QTLs), so it is not simple to improve them. Also, the crop environment varies depending on the season, year, location, and a specific area within the field. These variations lead to large fluctuations in crop yield. Therefore, breeders need to assess yield stability of crops in different environments to develop and launch a new variety.

Recent advances in rice genomics have improved our understanding of individual QTL functions, thereby unraveling genotype-phenotype relationships and facilitating grain yield improvement [8, 9, 10, 11]. Despite these, very little is known about the QTLs associated with biomass yield at maturity stage. QTL mapping studies on biomass yield [6, 12, 13] have not been widely publicized as grain yield is generally considered to be a priority.

In a previous study, we identified six QTLs that were responsible for >40% of the phenotypic variance in the total plant weight of recombinant inbred lines (RILs) of the high-yielding Japanese rice cultivars ‘Tachisugata’ (mainly for WCS) and ‘Hokuriku 193’ (primarily for grain, but also for WCS) [14]. The six QTLs were mapped on chromosomes 1, 2, 3, 5 and 10. At least in two QTLs, the ‘Tachisugata’ alleles displayed positive effects, whereas in the other four QTLs, the ‘Hokuriku 193’ alleles had a positive effect. The favorable combination of these QTL alleles resulted in a significant increase in plant weight compared to that of their parents, suggesting that QTL-based selection is effective for improving plant weight [14]. However, as this study covered only a single year, it remained unclear whether the QTL alleles would have consistent effects on plant weight over multiple years or in multiple environments. To address this question and to learn about the components of phenotypic variance, we designed multiple-environment experiments using the same set of RILs.

## Materials and methods

### Plant materials and growth conditions

The 188 RILs were derived from a cross between ‘Tachisugata’ (TS) and ‘Hokuriku 193’ (H193) [14–16]. For phenotype analysis, the parental cultivars and RILs were grown during the summer in an experimental paddy field at the Institute of Crop Science, NARO in Tsukubamirai (latitude 36°02′N, longitude 140°04′E), Japan, with 3 rows of 14 plants in each plot, in 6 environments (Table 1, S1 Fig). Seeds were sown in late April, and 30-d-old seedlings were transplanted into the field at 15 cm between plants and 30 cm between rows.

**Table 1:** Summary of experimental environments.

Env.	Year	Fertilization (N, P, K)	Generation of RILs
E1	2011	80 kg/ha each	F_6_
E2	2011	80 kg/ha each	F_6_
E3	2012	80 kg/ha each	F_7_
E4	2012	120 kg/ha each	F_7_
E5	2013	80 kg/ha each	F_8_
E6	2014	80 kg/ha each	F_9_

E1 and E2 were experimental replications of the environments, but they were two distinct blocks divided by a ridge. Controlled release fertilizer (LP100) was applied as the basal fertilizer in all the environments. LP100 releases about 80% of the total nitrogen content at a uniform rate for up to 100 days after application at 25°C. In E3, a quick-acting fertilizer was added as topdressing (40 kg/ha each for N, P, and K) at 30 days after transplanting. Soil type was clay loam. In environments E1, E3, E5, and E6, the RILs were grown in the same block.

### Phenotype analysis

As described in a previous study [14], we evaluated three biomass-related traits, namely: plant weight (PW, g, aboveground parts only), grain weight (GW, g), and stem and leaf weight (SLW, g), where PW = GW + SLW. Materials from 10 mature plants were bulked, dried for 48 h at 80°C, and weighed. To eliminate field border effects, only the 10 plants in the center of the middle row of each plot were used.

### Genotype analysis

In order to evaluate the genotype of the RILs, we genotyped the F_7_ lines of the RILs (the F_5_ lines were genotyped in a previous study [14]). We extracted genomic DNA from 2-month-old seedlings using the cetyltrimethylammonium bromide (CTAB) method [17]. Then we selected 384 single nucleotide polymorphisms (SNPs) between TS and H193 (192 SNPs were adopted from a previous study [14]) from a published SNP data set in the Q-TARO database (http://qtaro.abr.affrc.go.jp/) [18, 19], and genotyped samples on a GoldenGate BeadArray platform (Illumina Inc., San Diego, CA, USA). Of the 384 SNPs, 363 were used for QTL analysis (S1 File).

### QTL analysis

The 188 RILs were used to construct a linkage map. The linkage order and genetic distances of the 363 marker loci were calculated in MAPMAKER/Exp v. 3.0 software [20], with residual heterozygotes considered as the missing data. QTL analysis was performed using composite interval mapping in WinQTL Cartographer v. 2.5 software [21] with a significance level of α = 0.05. QTLs were added using forward and backward regression with the standard model (model 6) for up to 5 control markers. A window size of 10 cM with a walk speed of 2 cM was used. Following permutation tests with 1000 replicates, significant LOD scores were assigned for each trait [22]. QTL analyses were performed for each environment using trait data obtained for that specific environment.

### Statistical analysis

Multiple regression analysis was performed using the six previously detected QTL alleles (AA01010927, Chr. 1, 40.7 Mb; AD02003294, Chr. 2, 10.0 Mb; ah03002520, Chr. 3, 35.1 Mb; AD05011295, Chr. 5, 27.0 Mb; aa10000954, Chr. 10, 4.0 Mb; and AA10003574, Chr. 10, 22.3 Mb) as independent variables, and the plant weights of the RILs as continuous variables (14). We replaced two of them with test00229 (34.8Mb on Chr 3) and K10sf005 (3.8Mb on Chr 10) on the basis of their physical positions, because different SNP typing arrays were used between the studies.

To test whether a QTL–environment interaction was involved in the phenotypic variance of RIL biomass-related traits, we performed a two-way analysis of variance (ANOVA), using the genotypes of SNPs nearest to the detected QTLs, in accordance with the following model:

$$V_{P}=V_{G}+V_{E}+V_{GE}$$

where *V*_*P*_ = phenotypic variance, *V*_*G*_ = genotypic variance, *V*_*E*_ = environmental variance, and *V*_*GE*_ = variance due to genotype × environment interaction [23]. To clarify the effects of the environment clearer, we evaluated independently by separating the QTL-environment interactions as QTL-year interactions (using the data from E1, E3, E5, and E6, i.e., across years) and QTL-fertilization interaction (using the data from E3 and E4, i.e., between fertilization conditions). Significance levels were corrected on the basis of a false discovery rate of α = 0.05 for multiple testing by trait, in accordance with the number of interaction tests [24].

Broad-sense heritability (*H*^2^ = *V*_*G*_/*V*_*P*_) was calculated for each trait using the data from all six environments [23].

All analyses were performed in JMP v. 9 software (SAS Institute Inc., Cary, NC, USA).

## Results and discussion

### Phenotypic variations in biomass-related traits in the RILs

**Fig 1:**
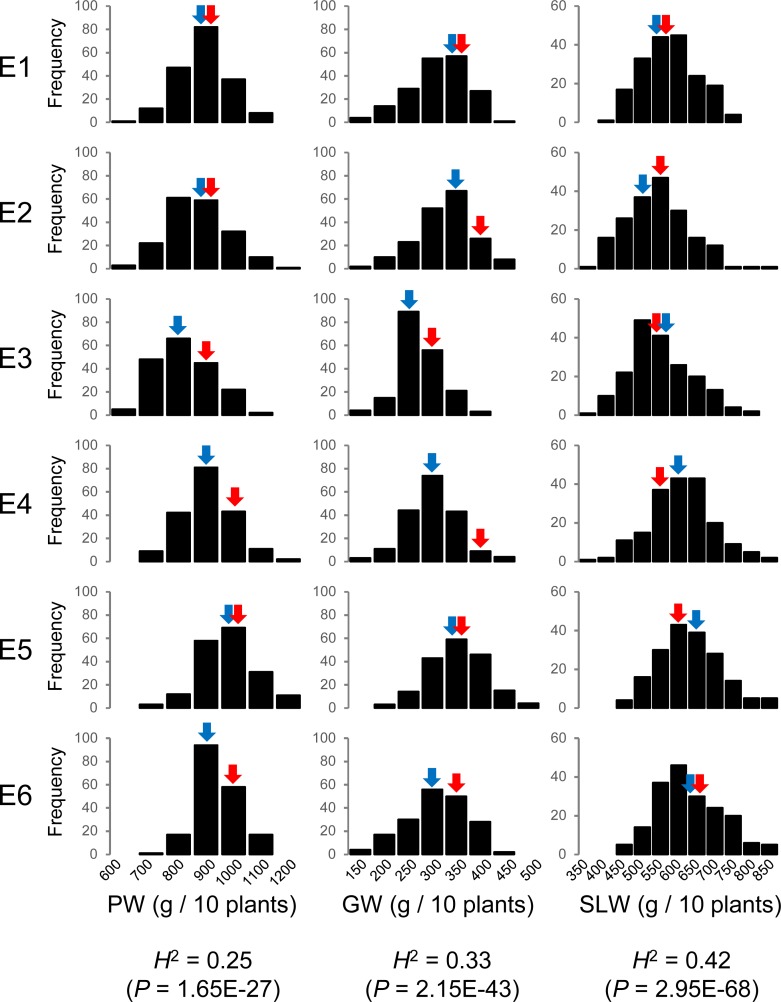
Frequency distributions and broad-sense heritabilities (*H*^2^) of biomass-related traits in RILs over multiple environments. Blue arrow, mean of ‘Tachisugata’; red arrow, mean of ‘Hokuriku 193’. E, environment; PW, plant weight; GW, grain weight; SLW, stem and leaf weight.

During the study period, the difference in mean temperature was small, but sunshine hours and solar radiation fluctuated widely among the different environments (S1 Fig). The mean PW and GW values of H193 were significantly greater than those of TS (*P* < 0.01) in some environments, whereas the mean SLW values of the parents were not significantly different (Fig 1, S1 Table). In the RILs, there was no obvious differences in the frequency distribution between E1 and E2 for all three traits. E1 and E2 were experimental replicates that were studies in the same year. However, the year-over-year fluctuations in the frequency distribution (E1, E3, E5, and E6) and those between the fertilization conditions (E3 and E4) were evident for all three traits (Fig 1, S1 Table). This suggests the influence of environmental effects on the phenotypic variations observed in rice [6, 25, 26], and the broad-sense heritabilities were all <0.50 (PW, 0.25; GW, 0.33; SLW, 0.42; Fig 1).

### Effects of the selected QTL alleles on biomass yield

**Fig 2:**
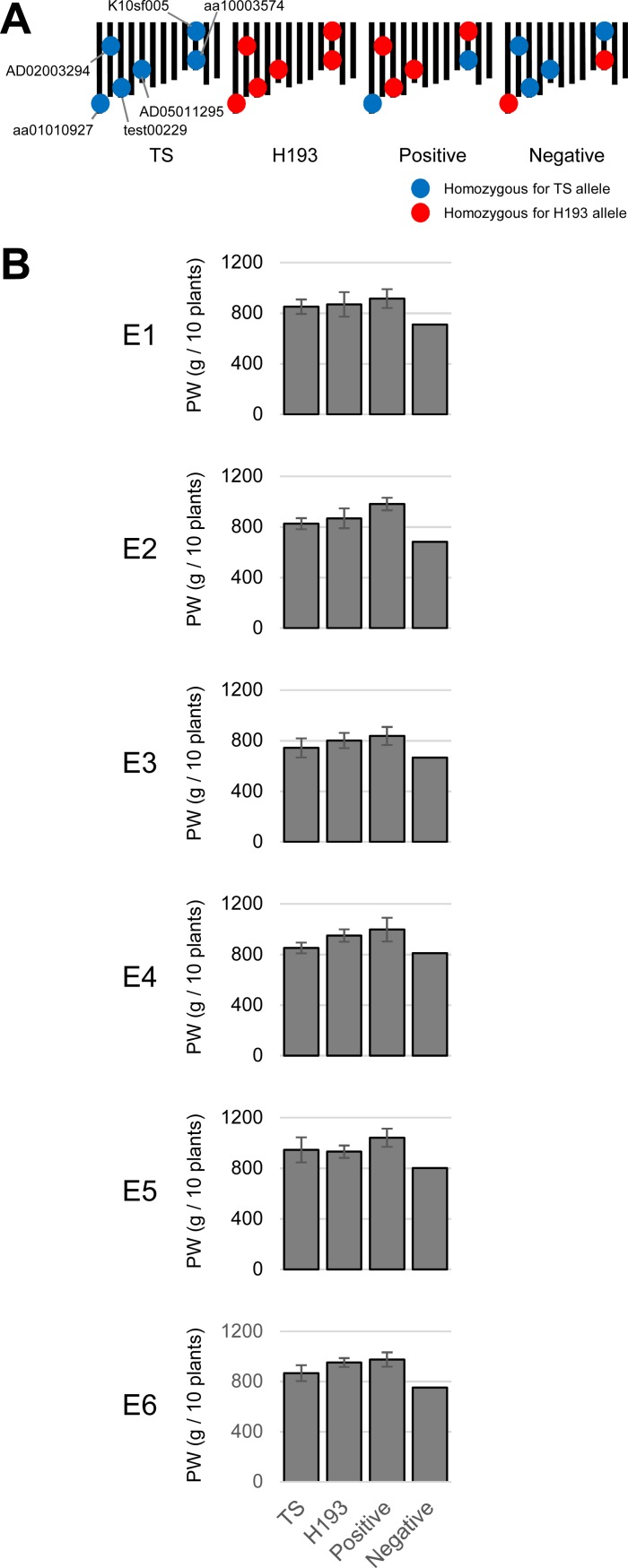
Effects of QTL alleles on plant weight. (A) Genotypes of parental cultivars and selected recombinant inbred lines (RILs). (B) Plant weights of parental cultivars (*n* = 7–9) and selected RILs. Values indicate means (± SD) of plant weights in each genotype class. Positive, RILs with QTL alleles with positive effect (*n* = 10); Negative, RILs with QTL alleles with negative effect (*n* = 2); PW, plant weight.

In a previous study, we used QTL mapping and multiple regression analysis to identify six QTLs that significantly contributed to PW [14]. The mean values of RILs with combinations of positive PW alleles were significantly higher than those of TS but similar to those of H193, and the mean values of RILs with combinations of negative PW alleles were significantly lower than those of both parental cultivars (Figs 2 and S2). Therefore, the selected genotypes showed a stable rank order of PW values across environments, although the frequency distribution of PW for all RILs fluctuated across the environments (Fig 1).

Multiple regression analysis revealed that the significance of the PW QTLs depended on environment. For example, a QTL on Chr. 1 (AA01010927) was stably significant across environments, whereas QTLs on Chr. 2 (AD02003294), Chr. 3 (test00229), Chr. 5 (AD05011295), and Chr. 10 (K10sf005 and AA10003574) were conditionally significant, dependent on environment. Therefore, the combined effects of the six QTL alleles on PW, as indicated by phenotypic variance explained (PVE), varied from 9.5% to 34.2%, which, except in E2 and E3, was similar to the broad-sense heritability of PW, showing that these six QTLs largely account for PW variations seen in the RILs (Fig 1, Table 2).

**Table 2:** Effects of previously detected QTLs on plant weight in multiple environments.

E	Partial regression coefficient						PVE (%)	*P*-value
	AA01010927	AD02003294	test00229	AD05011295	K10sf005	AA10003574		
	Chr. 1	Chr. 2	Chr. 3	Chr. 5	Chr. 10	Chr. 10		
	40.7 Mb	10.0 Mb	34.8 Mb	27.0 Mb	3.8 Mb	22.3 Mb		
E1	0.248***	−0.334***	−0.198**	−0.173*	−0.143*	0.212**	26.6	9.12E-10
E2	0.249***	−0.350***	−0.177*	−0.111^​^^*n*.*s*^^.^	−0.234***	0.309***	34.2	3.26E-13
E3	0.200*	−0.033^​^^*n*.*s*^^.^	−0.051^​^^*n*.*s*^^.^	−0.177*	−0.166*	0.129^​^^*n*.*s*^^.^	9.5	1.62E-03
E4	0.328***	−0.066^​^^*n*.*s*^^.^	−0.133^​^^*n*.*s*^^.^	−0.270***	−0.153*	0.177*	23.2	2.33E-08
E5	0.384***	−0.158*	−0.021^​^^*n*.*s*^^.^	−0.353***	−0.139^​^^*n*.*s*^^.^	0.099^​^^*n*.*s*^^.^	27.8	4.25E-10
E6	0.340***	−0.024^​^^*n*.*s*^^.^	−0.185*	−0.262***	−0.131^*​*^^*n*.*s*^^.^	0.152*	22.3	5.75E-08

Trait values were standardized before analysis, and the closest markers to the QTLs were used as independent variables. E, environment; PVE, phenotypic variance explained (adjusted *R*^2^-value × 100)

*0.01 < *P* < 0.05

**0.001 < *P* < 0.01

***P < 0.001

*n*.*s*., not significant.

To partition the variance of PW, we performed a two-way ANOVA using the data from E1, E3, E5, and E6 (across years). The analysis indicated that the contributions of environmental and genotypic variances to the variance of PW were highly significant (α < 0.05; Table 3), whereas that of the QTL–environment (Q–E) interaction was marginally significant (*P* = 0.04, but not significant at α < 0.05) only for the QTL on Chr. 2 (AD02003294) across years. Furthermore, the contribution of the Q–E interaction to PW variance was estimated to be subtle, owing to the small difference (0.51%) in PVEs between the additive (*V*_*P*_ = *V*_*G*_ + *V*_*E*_) and full (*V*_*P*_ = *V*_*G*_ + *V*_*E*_ + *V*_*GE*_) models. No significant Q-E interaction (even marginal) was detected between the fertilization conditions (E3 and E4) (S2 Table).

**Table 3:** Effects of QTLs, environments, and Q–E interactions on plant weight across years.

Marker analyzed	Chr.	QTL (*d*.*f*. = 1)		E (*d*.*f*. = 3)		QTL–E (*d*.*f*. = 3)	
		*F*-value	*P*-value	*F*-value	*P*-value	*F*-value	*P*-value
AA01010927	1	68.0	7.84E-16	100.5	2.14E-54	1.2	*n*.*s*.
AD02003294	2	12.0	5.71E-04	82.8	5.10E-46	2.9	4.01E-02
test00229	3	3.6	*n*.*s*.	96.0	2.90E-52	2.4	*n*.*s*.
AD05011295	5	28.6	1.22E-07	104.0	7.54E-56	1.2	*n*.*s*.
K10sf005	10	11.8	6.23E-04	95.4	4.46E-52	0.2	*n*.*s*.
AA10003574	10	34.1	7.81E-09	102.8	2.91E-55	0.5	*n*.*s*.

Chr., chromosome; E, environment; QTL–E, QTL–environment interaction; *d*.*f*., degrees of freedom; *n*.*s*., not significant at α = 0.05.

### Multiple-environment QTL mapping followed by analysis of components of PW variance

The contribution of Q–E interaction to PW variance was likely small in the selected QTLs. So we assessed its contribution in all of the significant QTLs detected by performing genome-wide multiple-environment QTL mapping.

**Fig 3:**
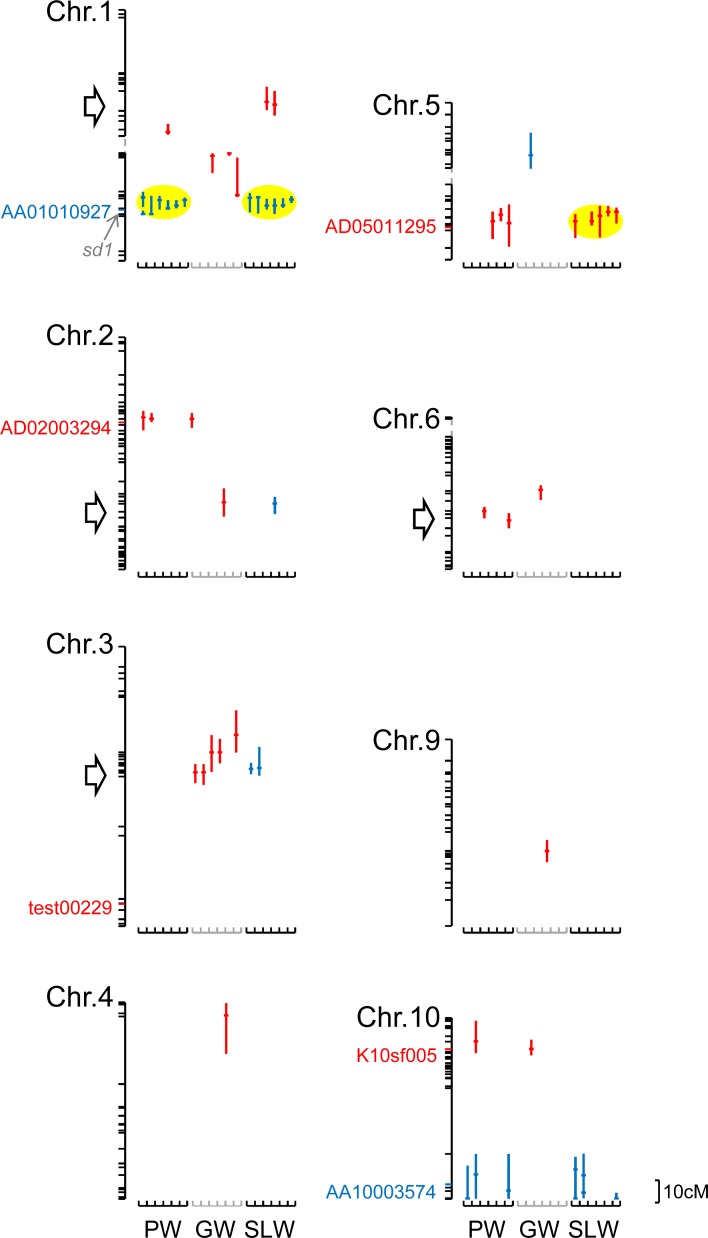
Locations of detected QTLs. Vertical bars to the right of the linkage maps denote 1-LOD confidence intervals, and horizontal bars denote the position of the LOD peak at each QTL. Color indicates whether ‘Tachisugata’ (blue) or ‘Hokuriku 193’ (red) alleles had a positive effect. The single nucleotide polymorphism (SNP) markers that were selected to increase biomass yield in our previous study are similarly colored. Yellow ellipses indicate stable or nearly stable QTLs; arrows indicate significant QTL regions detected in the previous study along with the six selected QTLs. Broken lines indicate linkage gaps. PW, plant weight; GW, grain weight; SLW, stem and leaf weight.

QTL analysis of PW, GW, and SLW was independently performed in each environment. We mapped at least 16 QTL regions for PW, GW, and SLW, where 1-LOD confidence intervals do not overlap with others from Chromosomes # 1–6, 9, and 10 (Fig 3, S3 Table), comprising 7 for PW (Chrs. 1, 2, 5, 6, 10), 9 for GW (Chrs. 1–6, 9, 10), and 6 for SLW (Chrs. 1–3, 5, 10). Of these, QTLs for PW and SLW (Chrs. 1, 10) explained ≥10% of the phenotypic variance in the traits of interest. A stable QTL for PW and SLW, which was significant across the environments, was located on Chr. 1 (38.5–41.0-Mb genomic region represented by AD01017981–ah01003209), and a nearly stable QTL for SLW, which was significant in five environments, was detected on Chr. 5 (24.1–25.3-Mb genomic region represented by K05sf166–Nag08KK11930; Fig 3, S3 Table). The significance of the other QTLs depended on environmental conditions (Fig 3, S3 Table).

The QTLs that were mapped around the 38.5–41.0-Mb genomic region of Chr. 1 explained >20% of the phenotypic variance of PW in E5 and of SLW in E5 and E6 (S3 Table, S3 Fig). The TS alleles had a positive effect. The QTLs putatively correspond to the *semi-dwarf 1* (*sd1*) locus, which is located at 40.1 Mb (Fig 3), as suggested previously [14, 27].

When we focused on the genomic regions around the six previously identified QTLs, we identified significant QTLs in five genomic regions by composite interval mapping, at least in some environments (Fig 3). However, no significant QTL was mapped in the genomic region around test00229 on Chr. 3. Instead, we detected several new QTLs for GW (on Chrs. 4, 5, and 9), although their significance was dependent on environment (Fig 3, S3 Table). Such results support the importance of follow-up experiments, as in the present study, for optimizing the markers used for QTL-based selection.

For all the QTLs detected in this study, we performed a two-way ANOVA across the years (E1, E3, E5, and E6) and between fertilization conditions (E3 and E4) in the same manner as used for the selected QTLs described above. The analysis indicated significant genotypic variance in almost all the QTLs and highly significant environmental variance for all QTLs, particularly for PW and SLW, despite controlling for a false discovery rate of α < 0.05 (S4 Table). On the other hand, no significant genotypic variance for all QTLs for GW and highly significant environmental variance for the all QTLs were detected (S4 Table). We also identified four significant Q–E interactions for GW at α < 0.05 (K01sf037 on Chr. 1, aa03002208 and ac03000537 on Chr. 3, and Nag08KK08918 on Chr. 4), and marginally significant Q–E interactions for PW (K05sf166 on Chr. 5), GW (test00058 on Chr. 1 and ah03001245 on Chr. 3), and SLW (P0686 and test00078 on Chr. 1; S4 Table). The difference in PVEs between the additive and full models in ANOVA was 1.59% for the GW QTL (ac03000537 that showed the largest *F*-value) and 0.54% for the PW QTL (K05sf166). Thus, the contribution of Q–E interaction to PW variance in all QTLs was estimated to be trivial across years. These results suggest that the response to environmental change was in large part parallel between TS and H193 alleles (or plasticity without Q–E interactions is a possibility) [28, 29]. No significant Q-E interaction (even marginal) in the three biomass-related traits was found between the fertilization conditions (E3 and E4) (S5 Table).

However, we cannot disregard the possibility that the contribution of Q–E interaction could be underestimated, owing to the limited number of RILs and of environmental conditions [Des Marais et al. 2013]. Indeed, under controlled environments, the presence of significant Q–E interaction has been often detected in rice studies [25, 30, 31]. Nevertheless, in an Experimental Station, year to year environmental fluctuations such as weather factors (e.g., temperature and sunshine) cannot be controlled or predicted in advance [32]. Such crucial information regarding the effects of QTLs and environment on target traits is important for genomics-based selection.

## Conclusions

Any scientific progress that improves the yield of rice will have a large effect on global food security and health. In our results, the biomass yield of all RILs fluctuated between years (environments; Fig 1). However, the rank order of plant weight of selected RILs that corresponded to their genotypes was consistently stable across environments (Fig 2). Some of the previously detected QTL alleles showed a stable effect on biomass yield in different environments. In addition, the fluctuation of biomass yield in all RILs appeared to be associated with the change of additive effects at QTLs in response to environmental change (that is, plasticity without Q–E interaction), rather than with Q–E interaction, although additional environmental settings at different locations and varying fertilization conditions will be useful to validate our findings. Therefore, these results show that QTL-based allele selection is helpful for the improvement of biomass yield in rice. In addition, as proposed by some researchers [33, 34], alleles detected in QTL analysis may provide further improvement by incorporation by more advanced selection methods (e.g., genomic selection) as fixed effects. Experimental approaches using such new selection methods will be focus of our future studies.

## Supporting information

S1 Fig Weather data in the growing period in six environments. E, environment.
(PPTX)

S2 Fig Effects of selected QTL alleles on plant weight in multiple environments. Values indicate means (± SD) of plant weights in each genotype class. Means of parental cultivars and recombinant inbred lines (RILs) in the six environments were compared by Tukey–Kramer HSD test. Positive, RILs with QTL alleles with positive effect; Negative, RILs with QTL alleles with negative effect; PW, plant weight.
(PPTX)

S3 Fig Locations of detected QTLs (a magnified version of Fig 3). Vertical bars to the right of the linkage maps denote 1-LOD confidence intervals, and horizontal bars denote the position of the LOD peak at each QTL. QTLs with PVE > 10% were shown by thick lines. Color indicates whether ‘Tachisugata’ (blue) or ‘Hokuriku 193’ (red) alleles had a positive effect. The single nucleotide polymorphism (SNP) markers that were selected to increase biomass yield in our previous study are similarly colored. Broken lines indicate linkage gaps. PW, plant weight; GW, grain weight; SLW, stem and leaf weight.
(PPTX)

S1 Table Phenotypic variation in the biomass-related traits of parental cultivars and RILs grown in multiple environments. The mean trait values of ‘Tachisugata’ and ‘Hokuriku 193’ were compared by a two-tailed Student’s *t*-test. PW, plant weight; GW, grain weight; SLW, stem and leaf weight; E, environment; *n*.*s*., not significant.
(XLSX)

S2 Table Effects of QTLs, environments, and Q–E interactions on plant weight between fertilization conditions. Chr, chromosome; E, environment; QTL-E, QTL-environment interaction; d.f., degrees of freedom; n.s., not significant at α = 0.05.
(XLSX)

S3 Table Summary of significant QTLs. Position is based on the physical position in the ‘Nipponbare’ rice (*O*. *sativa* ssp. *japonica*) genome (Build 4, http://rgp.dna.affrc.go.jp/). AE (additive effect) indicates the effect of the ‘Tachisugata’ allele relative to that of the ‘Hokuriku 193’ allele. E, environment; Chr., chromosome; PVE, phenotypic variance explained (= *R*^2^-value × 100); PW, plant weight; GW, grain weight; SLW, stem and leaf weight.
(XLSX)

S4 Table Effects of QTLs, environments, and Q–E interactions on biomass-related traits across years. Chr., chromosome; E, environment; QTL–E, QTL–environment interaction. PW, plant weight; GW, grain weight; SLW, stem and leaf weight. **Boldface** indicates significance at α = 0.05. *n*.*s*., not significant at α = 0.05. Additive, additive model (*V*_*P*_ = *V*_*Q*_ + *V*_*E*_). Full, full model (*V*_*P*_ = *V*_*Q*_ + *V*_*E*_ + V_*QE*_).
(XLSX)

S5 Table Effects of QTLs, environments, and Q–E interactions on biomass-related traits between fertilization conditions. Chr., chromosome; E, environment; QTL–E, QTL–environment interaction. PW, plant weight; GW, grain weight; SLW, stem and leaf weight. **Boldface** indicates significance at α = 0.05. *d*.*f*., degrees of freedom. *n*.*s*., not significant at α = 0.05. Additive, additive model (*V*_*P*_ = *V*_*Q*_ + *V*_*E*_). Full, full model (*V*_*P*_ = *V*_*Q*_ + *V*_*E*_ + *V*_*QE*_).
(XLSX)

S1 File Genotype and phenotype data. (XLSX)
