# Supplementary material for: A follow-up study for biomass yield QTLs in rice
Source: PLoS One. 2018 Oct 23;13(10):e0206054. doi: 10.1371/journal.pone.0206054 (PMC6198978; doi:10.1371/journal.pone.0206054)
Supplement: S1 Fig — E, environment. (PPTX) [file pone.0206054.s001.pptx]

## Slide 1
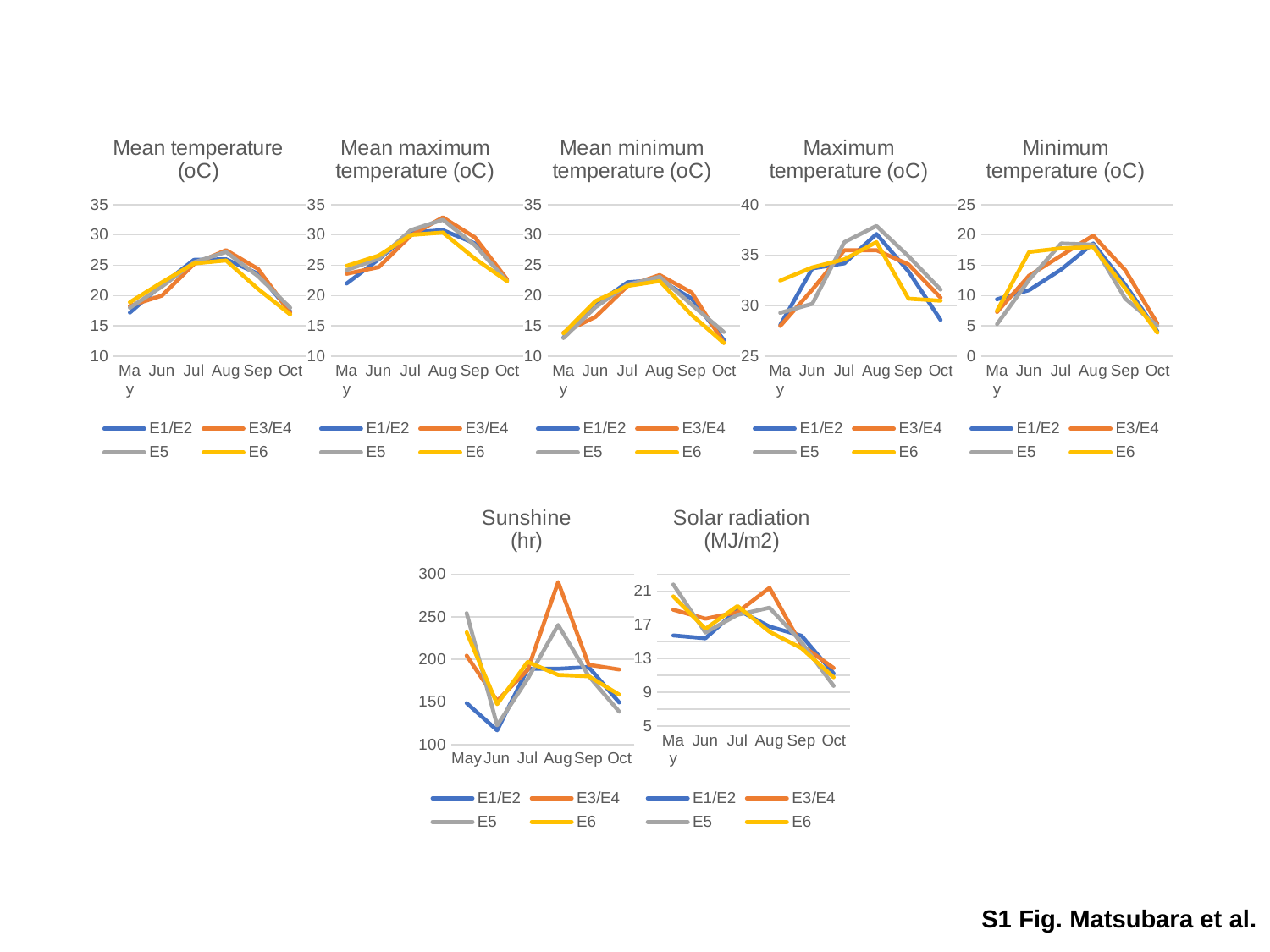

### Chart: Mean temperature (oC)
| Category | E1/E2 | E3/E4 | E5 | E6 |
|---|---|---|---|---|
| May | 17.2 | 18.3 | 18.0 | 18.9 |
| Jun | 21.8 | 20.0 | 21.5 | 22.2 |
| Jul | 25.9 | 25.2 | 25.5 | 25.3 |
| Aug | 26.0 | 27.5 | 27.2 | 25.8 |
| Sep | 23.6 | 24.4 | 23.2 | 21.1 |
| Oct | 17.4 | 17.2 | 18.0 | 16.9 |
### Chart: Mean maximum temperature (oC)
| Category | E1/E2 | E3/E4 | E5 | E6 |
|---|---|---|---|---|
| May | 22.0 | 23.6 | 24.2 | 24.9 |
| Jun | 26.0 | 24.7 | 26.1 | 26.6 |
| Jul | 30.4 | 29.9 | 30.8 | 30.0 |
| Aug | 30.8 | 32.9 | 32.5 | 30.4 |
| Sep | 28.6 | 29.6 | 28.3 | 26.1 |
| Oct | 22.5 | 22.7 | 22.4 | 22.4 |
### Chart: Mean minimum temperature (oC)
| Category | E1/E2 | E3/E4 | E5 | E6 |
|---|---|---|---|---|
| May | 13.1 | 13.9 | 13.0 | 13.8 |
| Jun | 18.5 | 16.5 | 18.1 | 19.1 |
| Jul | 22.2 | 21.6 | 21.8 | 21.6 |
| Aug | 22.6 | 23.4 | 23.1 | 22.4 |
| Sep | 19.5 | 20.5 | 18.5 | 16.8 |
| Oct | 12.7 | 12.2 | 14.0 | 12.2 |
### Chart: Maximum temperature (oC)
| Category | E1/E2 | E3/E4 | E5 | E6 |
|---|---|---|---|---|
| May | 28.1 | 28.0 | 29.3 | 32.5 |
| Jun | 33.7 | 31.6 | 30.2 | 33.8 |
| Jul | 34.2 | 35.5 | 36.3 | 34.6 |
| Aug | 37.1 | 35.5 | 37.9 | 36.3 |
| Sep | 33.4 | 34.1 | 34.9 | 30.7 |
| Oct | 28.6 | 30.8 | 31.6 | 30.5 |
### Chart: Minimum temperature (oC)
| Category | E1/E2 | E3/E4 | E5 | E6 |
|---|---|---|---|---|
| May | 9.4 | 7.3 | 5.3 | 7.5 |
| Jun | 10.9 | 13.3 | 12.6 | 17.2 |
| Jul | 14.3 | 16.6 | 18.6 | 17.8 |
| Aug | 18.6 | 19.9 | 18.4 | 18.0 |
| Sep | 11.8 | 14.2 | 9.5 | 11.2 |
| Oct | 4.1 | 5.4 | 5.0 | 3.9 |
### Chart: Sunshine
(hr)
| Category | E1/E2 | E3/E4 | E5 | E6 |
|---|---|---|---|---|
| May | 148.5 | 204.4 | 254.3 | 231.8 |
| Jun | 116.6 | 151.2 | 122.4 | 147.3 |
| Jul | 188.8 | 187.5 | 177.1 | 197.0 |
| Aug | 188.9 | 290.8 | 240.4 | 181.7 |
| Sep | 190.9 | 193.6 | 180.4 | 180.1 |
| Oct | 149.3 | 188.0 | 138.5 | 158.5 |
### Chart: Solar radiation
(MJ/m2)
| Category | E1/E2 | E3/E4 | E5 | E6 |
|---|---|---|---|---|
| May | 15.742 | 18.797 | 21.79 | 20.376 |
| Jun | 15.402 | 17.724 | 16.089 | 16.524 |
| Jul | 18.797 | 18.495 | 18.183 | 19.229 |
| Aug | 16.789 | 21.383 | 19.031 | 16.181 |
| Sep | 15.706 | 14.593 | 14.937 | 14.222 |
| Oct | 11.226 | 11.864 | 9.75 | 10.774 |S1 Fig. Matsubara et al.
