# Supplementary material for: A follow-up study for biomass yield QTLs in rice
Source: PLoS One. 2018 Oct 23;13(10):e0206054. doi: 10.1371/journal.pone.0206054 (PMC6198978; doi:10.1371/journal.pone.0206054)
Supplement: S2 Fig — Values indicate means (± SD) of plant weights in each genotype class. Means of parental cultivars and recombinant inbred lines (RILs) in the six environments were compared by Tukey–Kramer HSD test. Positive, RILs with QTL alleles with positive effect; Negative, RILs with QTL alleles with negative effect; PW, plant weight. (PPTX) [file pone.0206054.s002.pptx]

## Slide 1
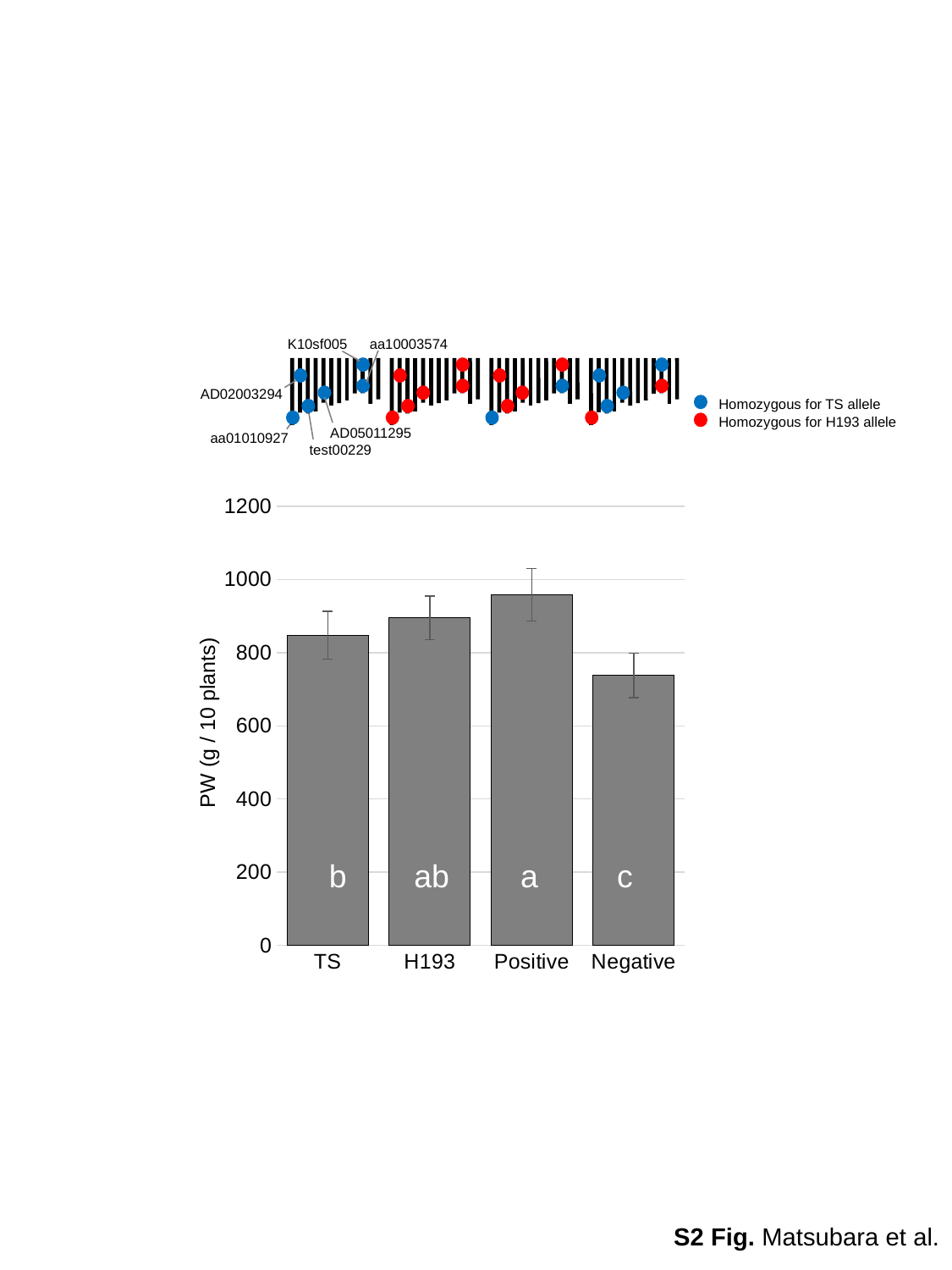

K10sf005
aa10003574
AD02003294
Homozygous for TS allele
Homozygous for H193 allele
AD05011295
aa01010927
test00229
### Chart
| Category | |
|---|---|
| TS | 847.6833333333333 |
| H193 | 895.1500000000001 |
| Positive | 958.2333333333335 |
| Negative | 737.5 |PW (g / 10 plants)
b
ab
a
c
S2 Fig. Matsubara et al.
