# Supplementary material for: A follow-up study for biomass yield QTLs in rice
Source: PLoS One. 2018 Oct 23;13(10):e0206054. doi: 10.1371/journal.pone.0206054 (PMC6198978; doi:10.1371/journal.pone.0206054)
Supplement: S3 Fig — Vertical bars to the right of the linkage maps denote 1-LOD confidence intervals, and horizontal bars denote the position of the LOD peak at each QTL. QTLs with PVE > 10% were shown by thick lines. Color indicates whether ‘Tachisugata’ (blue) or ‘Hokuriku 193’ (red) alleles had a positive effect. The single nucleotide polymorphism (SNP) markers that were selected to increase biomass yield in our previous study are similarly colored. Broken lines indicate linkage gaps. PW, plant weight; GW, grain weight; SLW, stem and leaf weight. (PPTX) [file pone.0206054.s003.pptx]

## Slide 1
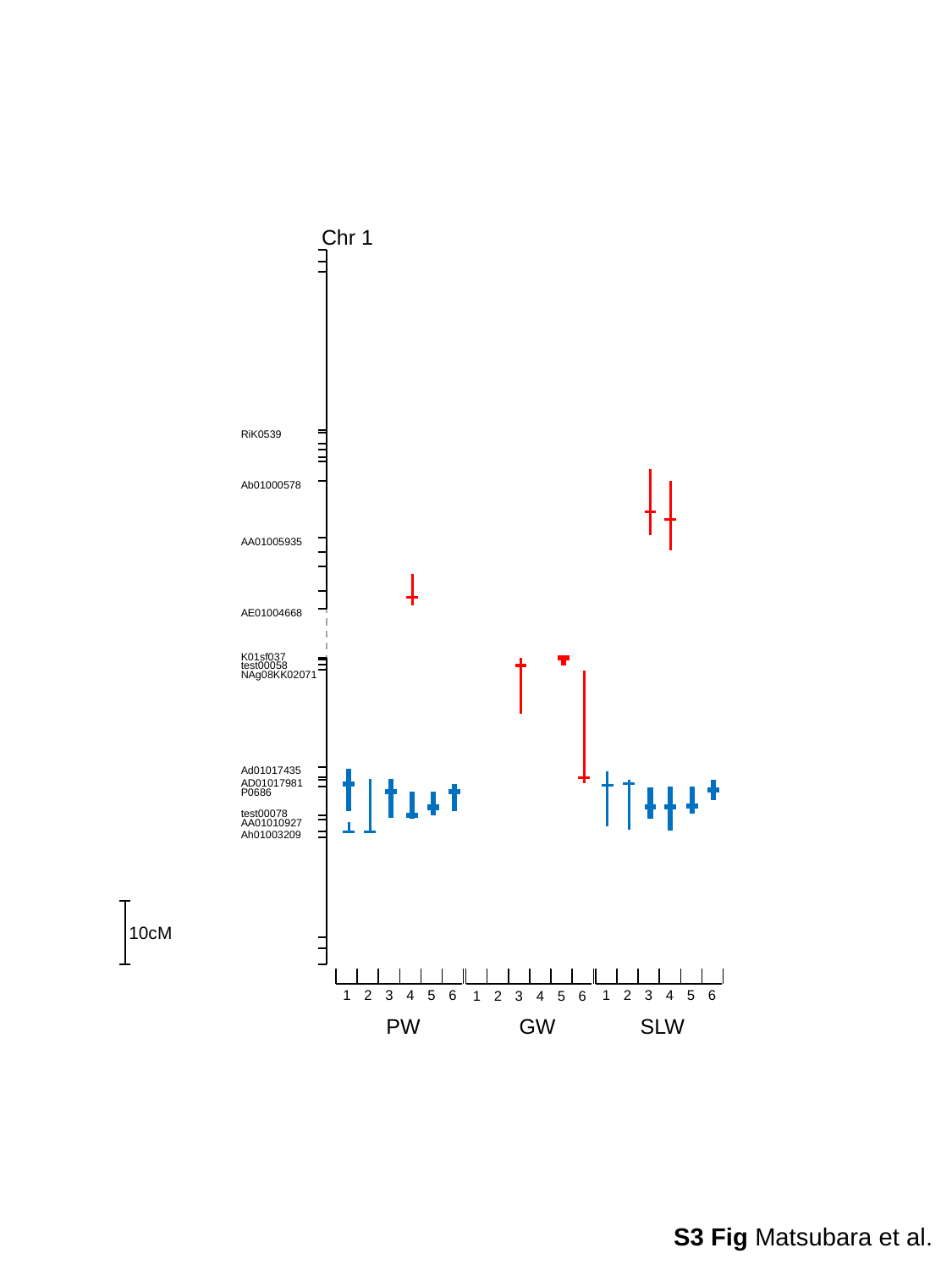

Chr 1
RiK0539
Ab01000578
AA01005935
AE01004668
K01sf037
test00058
NAg08KK02071
Ad01017435
AD01017981
P0686
test00078
AA01010927
Ah01003209
10cM
1
2
3
4
5
6
1
2
3
4
5
6
1
2
3
4
5
6
PW
SLW
GW
S3 Fig Matsubara et al.

## Slide 2
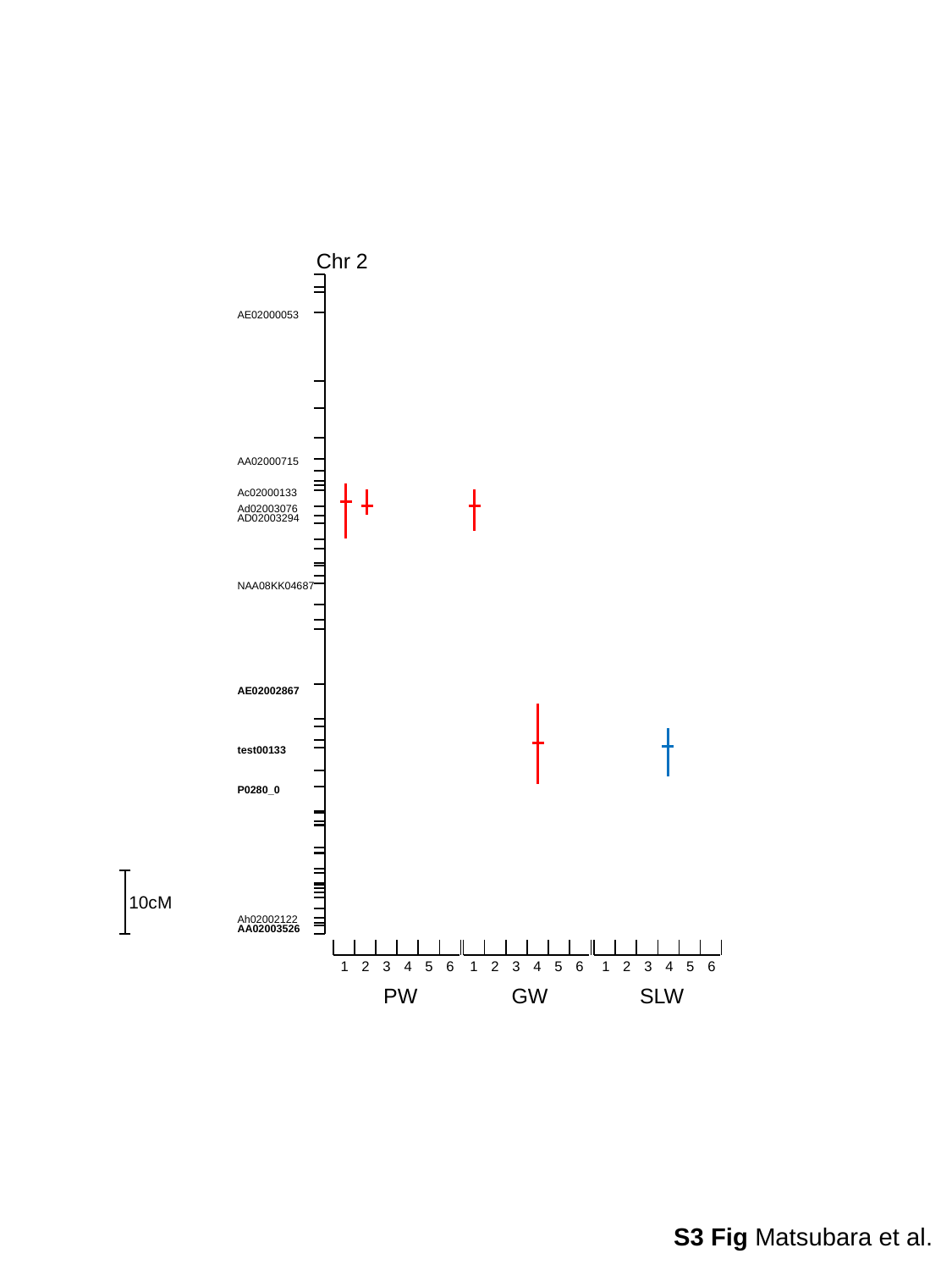

Chr 2
AE02000053
AA02000715
Ac02000133
Ad02003076
AD02003294
NAA08KK04687
AE02002867
test00133
P0280_0
10cM
Ah02002122
AA02003526
1
2
3
4
5
6
1
2
3
4
5
6
1
2
3
4
5
6
PW
GW
SLW
S3 Fig Matsubara et al.

## Slide 3
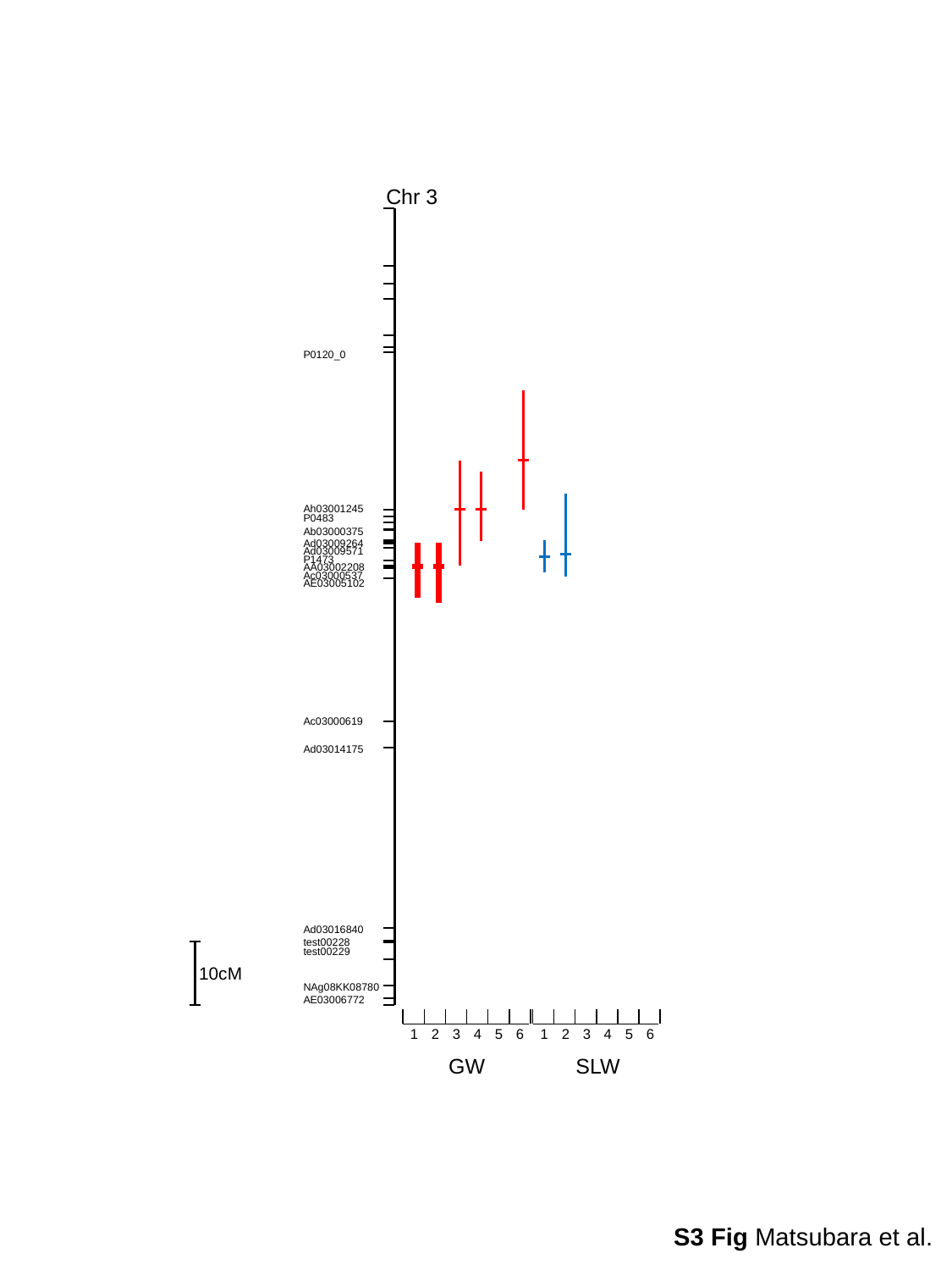

Chr 3
P0120_0
Ah03001245
P0483
Ab03000375
Ad03009264
Ad03009571
P1473
AA03002208
Ac03000537
AE03005102
Ac03000619
Ad03014175
Ad03016840
test00228
10cM
test00229
NAg08KK08780
AE03006772
1
2
3
4
5
6
1
2
3
4
5
6
GW
SLW
S3 Fig Matsubara et al.

## Slide 4
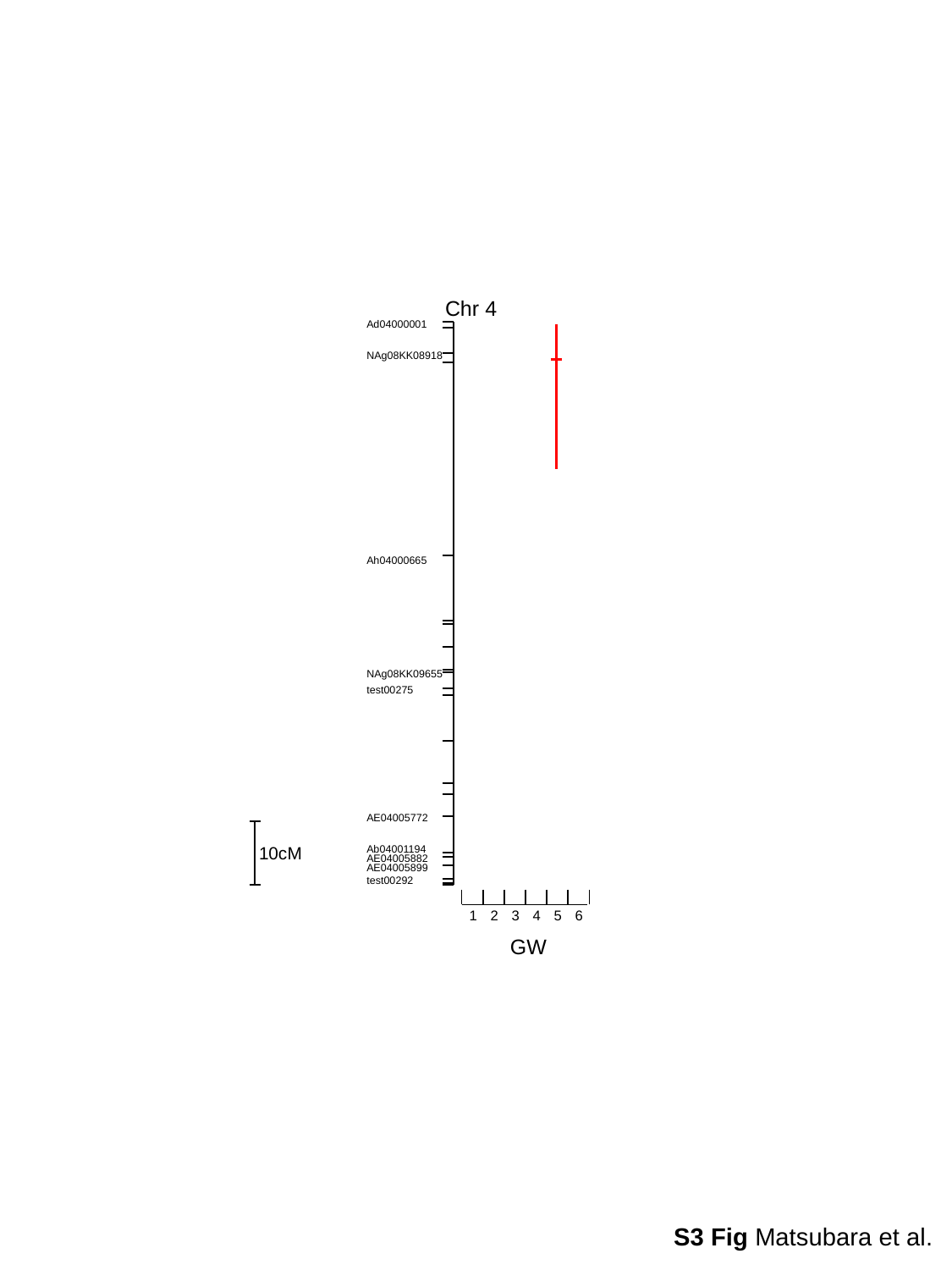

Chr 4
Ad04000001
NAg08KK08918
Ah04000665
NAg08KK09655
test00275
AE04005772
10cM
Ab04001194
AE04005882
AE04005899
test00292
1
2
3
4
5
6
GW
S3 Fig Matsubara et al.

## Slide 5
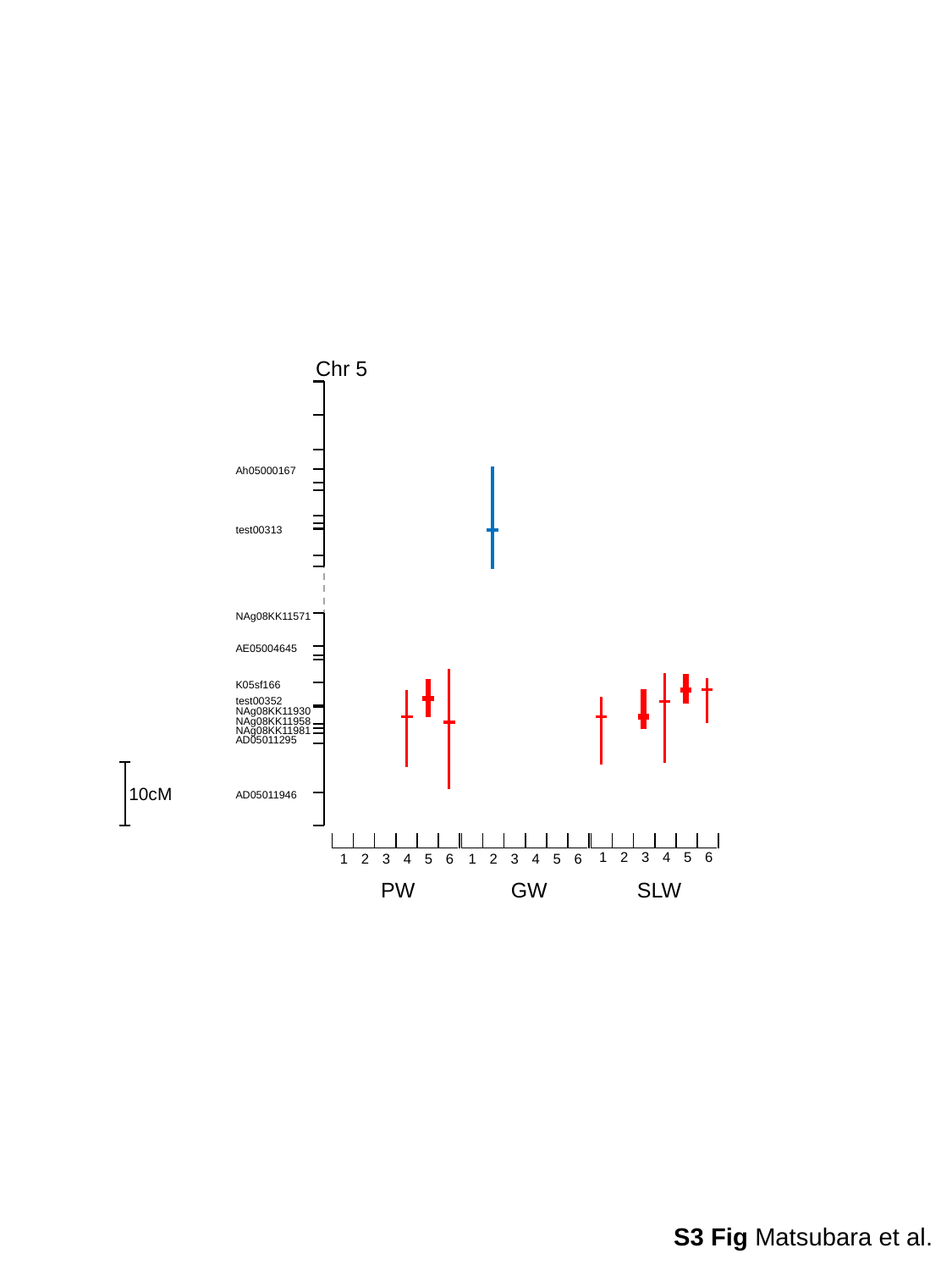

Chr 5
Ah05000167
test00313
NAg08KK11571
AE05004645
K05sf166
test00352
NAg08KK11930
NAg08KK11958
NAg08KK11981
AD05011295
10cM
AD05011946
1
2
3
4
5
6
1
2
3
4
5
6
1
2
3
4
5
6
PW
GW
SLW
S3 Fig Matsubara et al.

## Slide 6
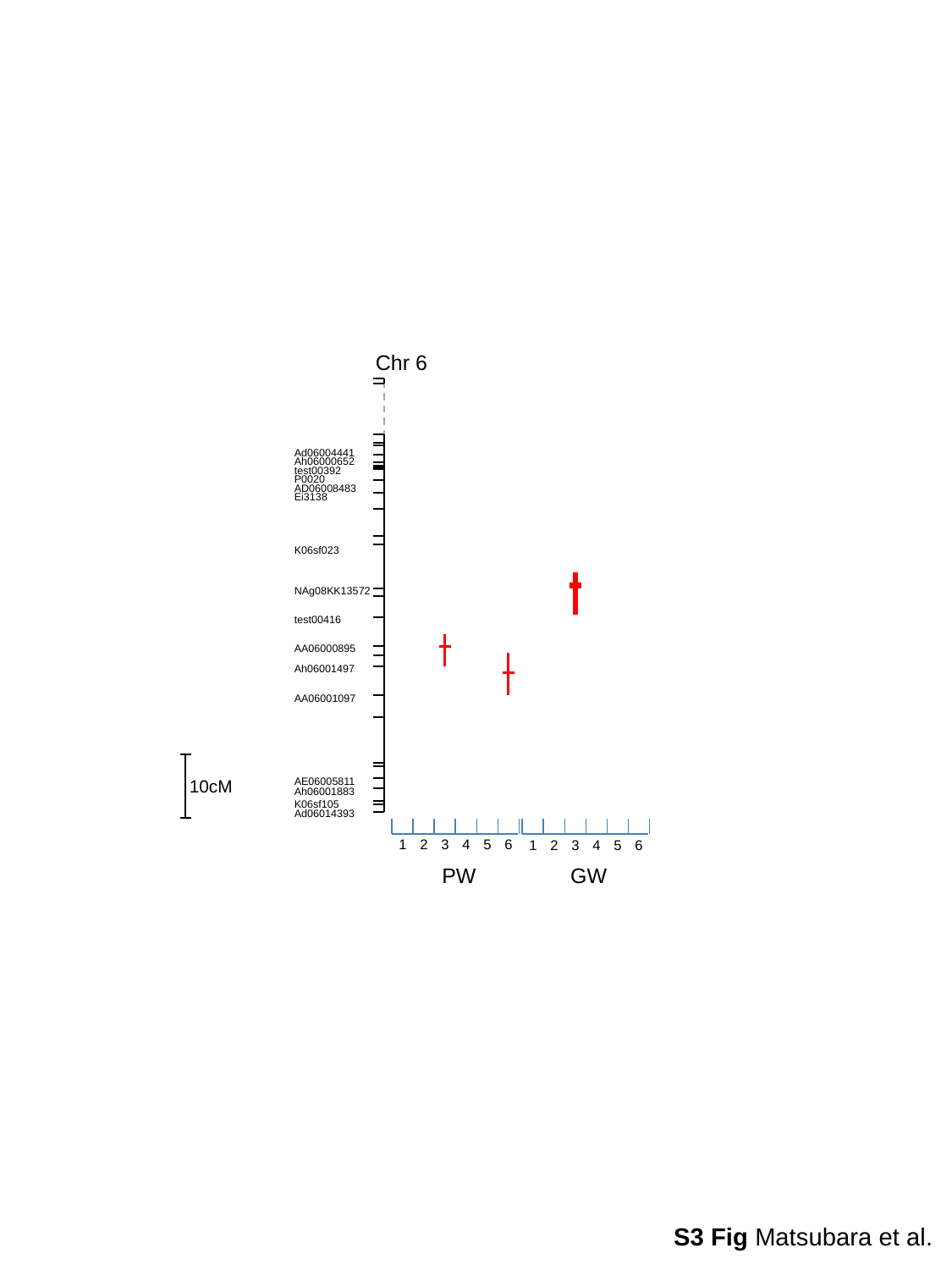

Chr 6
Ad06004441
Ah06000652
test00392
P0020
AD06008483
Ei3138
K06sf023
NAg08KK13572
test00416
AA06000895
Ah06001497
AA06001097
10cM
AE06005811
Ah06001883
K06sf105
Ad06014393
1
2
3
4
5
6
1
2
3
4
5
6
PW
GW
S3 Fig Matsubara et al.

## Slide 7
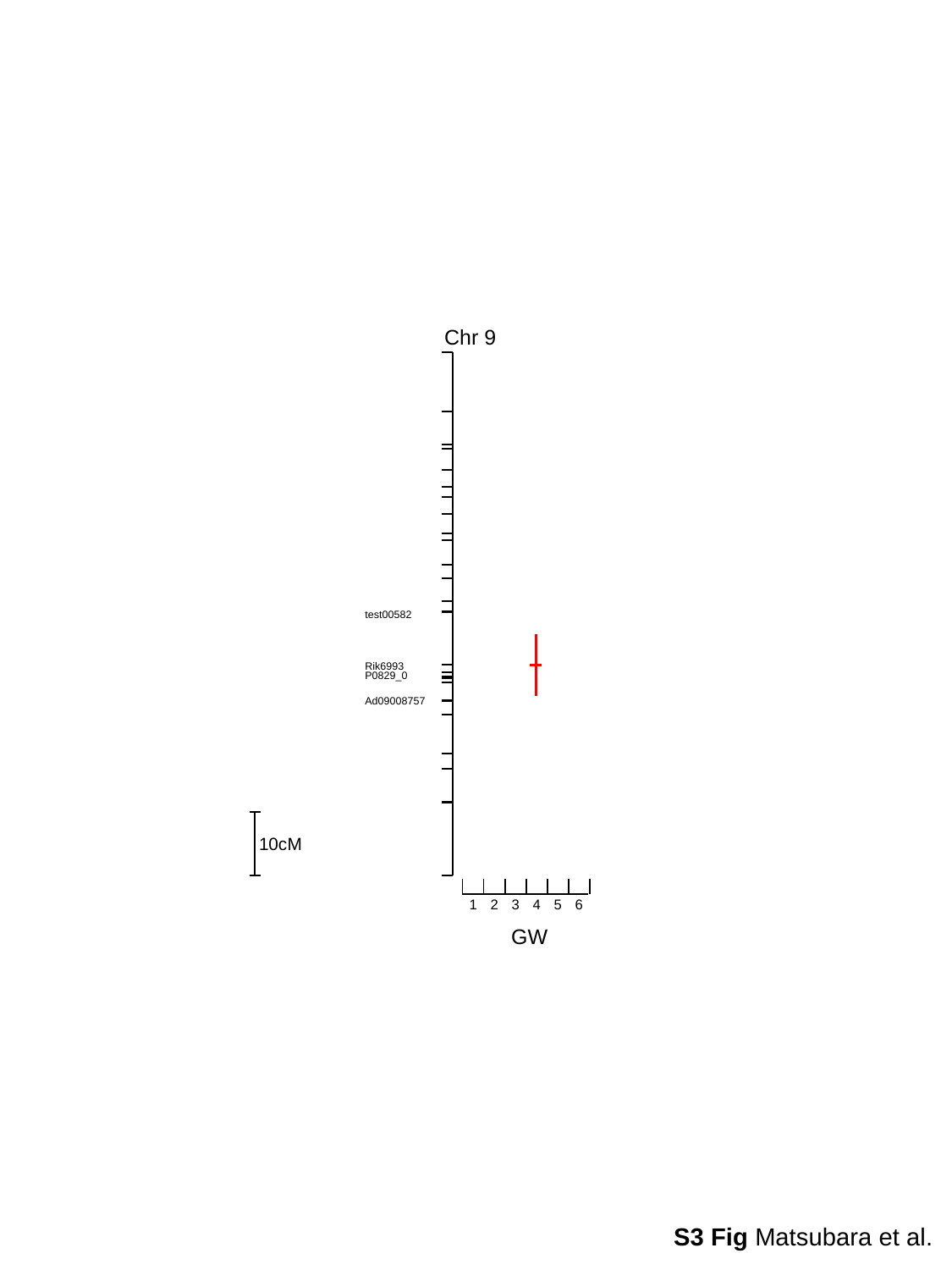

Chr 9
test00582
Rik6993
P0829_0
Ad09008757
10cM
1
2
3
4
5
6
GW
S3 Fig Matsubara et al.

## Slide 8
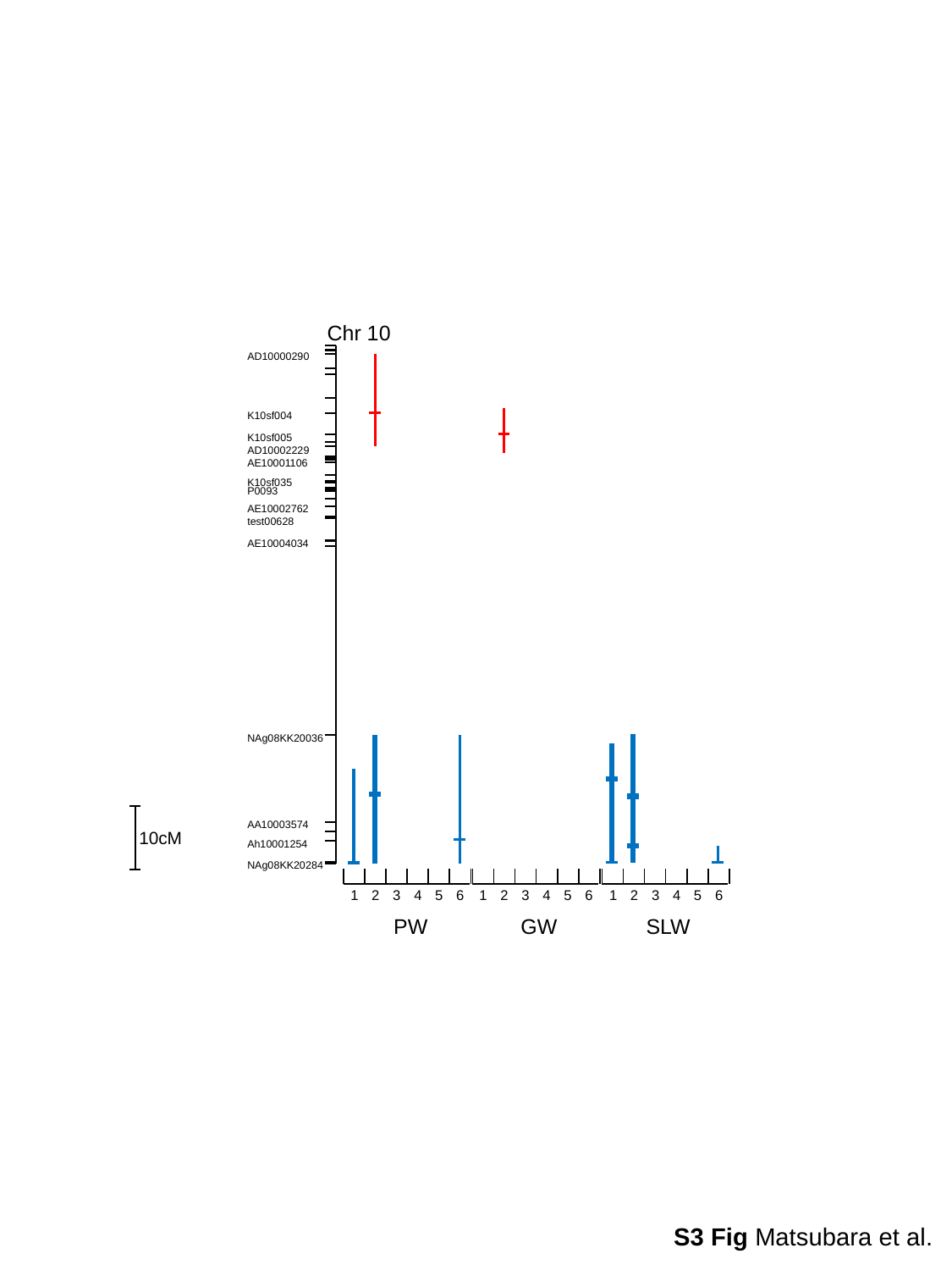

Chr 10
AD10000290
K10sf004
K10sf005
AD10002229
AE10001106
K10sf035
P0093
AE10002762
test00628
AE10004034
NAg08KK20036
10cM
AA10003574
Ah10001254
NAg08KK20284
1
2
3
4
5
6
1
2
3
4
5
6
1
2
3
4
5
6
PW
GW
SLW
S3 Fig Matsubara et al.
